# Intrapleural injection of urokinase in the treatment of acute *Haemophilus influenza* empyema in children: A case report and literature review

**DOI:** 10.3389/fped.2022.882005

**Published:** 2022-07-22

**Authors:** Lin Yang, YaFei Zhu, GuangSheng Wu

**Affiliations:** Department of Pediatrics, The Affiliated Hospital of Hangzhou Normal University, Hangzhou, China

**Keywords:** *Haemophilus influenzae*, empyema, urokinase, high-throughput, infection

## Abstract

**Objective:**

The purpose of this study is to analyze the clinical data of a child with acute empyema caused by *Haemophilus influenzae*, and to investigate the diagnosis and treatment of this disease through literature review to improve the clinical understanding of this kind of disease.

**Methods:**

A 6-year-old female with acute *H. influenzae* empyema was treated at the Department of Pediatrics of The Affiliated Hospital of Hangzhou Normal University, Hangzhou, China. The pleural puncture fluid turned out to be yellow turbid pus, and the pleural effusion was diagnosed as empyema according to the classification of pleural effusions. High-throughput sequencing revealed the presence of *H. influenzae*. After comprehensive treatment, including antibiotics, closed pleural drainage, and intrapleural injection of urokinase, the pleural effusion was absorbed and discharged. A systematic literature search in Pubmed, Embase, Scopus, CNKI, Wanfang, and VIP Chinese databases revealed no cases of acute empyema in children caused by *H. influenza* and treated with urokinase.

**Results:**

There was no bronchopleural fistula and tension pneumothorax during the treatment. One month after discharge, chest computed tomography (CT) revealed no pleural thickening and normal pulmonary function.

**Conclusion:**

Pneumonia in the child worsened after an initial improvement of symptoms, which is an issue that requires further medical attention. High-throughput sequencing of pathogens in pleural effusion can improve the detection rate. This study indicated that closed pleural drainage combined with intrapleural injection of urokinase is an effective treatment for *H. influenzae* empyema in children.

## Introduction

*Haemophilus influenzae* (*H*. i*nfluenzae*) is one of the important pathogens in pediatric community-acquired pneumonia, and a small proportion of *H*. *influenzae* infections are bacteremic pneumonia with severe systemic symptoms and can be combined with acute septic chest (1, 2). The key to the treatment of pleural empyema is to effectively drain the pus from the chest cavity and eliminate the residual pleural cavity (3, 4). We now report a case of acute *H. influenza* pleural empyema in a child treated with an intrapleural injection of urokinase. Intrathoracic injection of urokinase after an ultrasound showed that there was a separation of pleural fluid and effective drainage of pleural fluid was achieved. By reviewing the relevant literature, it is hoped that pediatricians will raise awareness of acute thorax abscess in the early stage of pulmonary infectious diseases, and once the diagnosis of thorax abscess is made, the possibility of H. *influenzae* infection needs to be considered to improve the clinical experience.

## Data and methods

### Study subjects

This study presents the case of a 6-year-old female patient diagnosed with the acute septic chest in the Department of Pediatrics, at The Affiliated Hospital of Hangzhou Normal University (Hangzhou, China), in January 2021. Informed consent was obtained from the patient's guardian for the collection of clinical information and invasive surgical procedures. The procedures in this study were in accordance with the requirements of the 2013 World Medical Association Declaration of Helsinki and approved by the hospital medical ethics committee (approval number: 2021(E2)-KS-025).

### Methods

#### Diagnostic criteria for acute septic pleural effusion in children

According to the diagnostic criteria of septic pleural effusion in Zhu Fu-Tang Practical Pediatrics, 8th edition, 2 of the following 3 criteria were met: (1) cell count >10 × 10^9^/L; (2) glucose ≤ 400 mg/L (2.2 mmol/L); and (3) lactate dehydrogenase ≥1,000 U/L.

#### Literature search strategy

A systematic literature search was performed in Pubmed, Embase, Scopus, CNKI, Wanfang, and VIP Chinese databases using “*Haemophilus influenzae*,” “septic chest,” “urokinase,” “*Haemophilus influenzae*,” “empyema,” and “urokinase” as keywords.

## Results

### Medical history collection

The patient, a female aged 6 years and 2 months, was admitted to the pediatric ward of The Affiliated Hospital of Hangzhou Normal University in January 2021, with “coughing and intermittent fever for 4 d and shortness of breath for 1 day.” The child had a maximum temperature of 40°C and a wet cough on the first d of illness. After 3 ds of antibiotic treatment with azithromycin in the Emergency Department for “pneumonia,” the fever subsided and the cough improved, but after 24 h, fever reappeared, the cough worsened, and the child developed shortness of breath. Therefore, the patient was admitted to our hospital for further treatment.

The child had previously undergone “atrial septal defect closure” at the Children's Hospital of Zhejiang University School of Medicine (Hangzhou, China) at the age of 1 year.

The patient had a physical examination on admission: her body temperature was 38°C, heart rate 140 beats/min, respiration 45 beats/min, and blood pressure of 117/81 mmHg. She had clear consciousness, irritability, shortness of breath, no perioral cyanosis, positive trigeminal sign, pharyngeal congestion, bilateral tonsil enlargement, no purulent secretions, and trachea in the center. The breath sounds were less audible on the left side with a dull percussion note, and the left side of the chest had a turbid percussion, while the right side had a clear sound. Additionally, bilateral rales were heard on auscultation. No tremor was detected in the precordial region. A grade 2/6 systolic murmur was heard between the 2nd and 3rd ribs at the left sternal border. There was no radiating pain in the precordial region and subxiphoid region; the whole abdominal muscle was tense, and the liver and spleen could not be palpated. The neurological examination was negative, and no rash over the whole body was observed during the clinical examination.

### Relevant examination results

#### Collected laboratory data

Blood routine: white blood cell counts 17.70 × 10^9^/L, neutrophil percentage 90.2%, red blood cell count 4.15 × 10^12^/L, hemoglobin 133 g/L, platelet count 296 × 109/L, and high-sensitivity C-reactive protein 314.37 mg/L. Respiratory virus antigen (nasal swab): negative for influenza A virus, influenza B virus, adenovirus, and respiratory syncytial virus antigen. IgM tests for respiratory pathogens are as follows: *Legionella pneumophila, Mycoplasma pneumoniae, Rickettsia pneumoniae, Chlamydia pneumoniae*, adenovirus, respiratory syncytial virus, influenza A virus, influenza B virus, and parainfluenza virus were all negative. Arterial blood gas analysis' results are as follows: blood pH 7.364, partial pressure of carbon dioxide 41.60 mmHg, partial pressure of oxygen 45.70 mmHg, oxygen saturation 81.90%, potassium 3.6 mmol/L, sodium 132 mmol/L, chloride 97 mmol/L, free calcium 1.18 mmol/L, lactate 0.9 mmol/L, actual residual base-1.7 mmol/ L, and standard residual base −1.5 mmol/L. T-cell test for tuberculosis infection is negative. Pleural and ascitic fluid biochemistry results are as follows: total protein 47 g/L, lactate dehydrogenase 977 U/L, adenosine deaminase 32.7 U/L, glucose 2.76 mmol/L, and amylase 22 U/L. Pleural fluid routine and cytological examination results are as follows: yellow color, turbid transparency, positive Rivalta test (++), nucleated cell count 21,643 /μl, and somatic red blood cell count 3,000/μl. Pleural fluid cytological classification examination results are as follows: 94% neutrophil classification, 1% lymphocyte classification, and 5% mononuclear macrophage classification. Pleural fluid *Mycoplasma pneumoniae* DNA test resulted negative for *Mycoplasma pneumoniae* DNA. Chest fluid tuberculosis smear examination showed no antacid *Bacilli* were detected in the smear. Pleural fluid Xpert MTB/RIF showed that no *Mycobacterium tuberculosis* complex group was detected. General bacterial culture of pleural fluid was negative. Pleural fluid anaerobic culture was negative. Pleural fluid *Haemophilus* culture was negative.

#### Nucleic acid detection revealed the following

**Figure 1:**
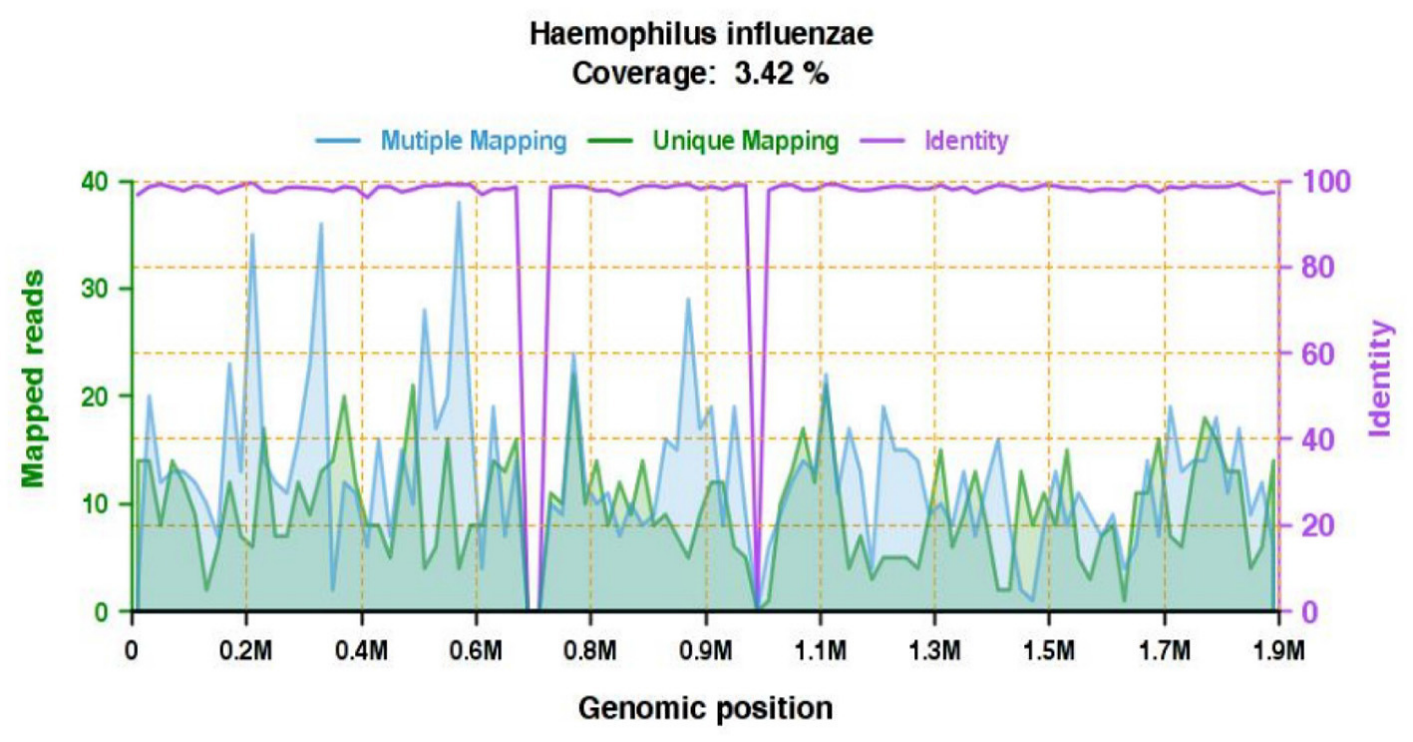
High confidence pathogen genome coverage map: gene coverage map of *H. influenzae*. The total number of bases in the genome of this species is 1,890,645 (bp) and the total length of the measured sequence coverage of this species is 64,657 (bp), with a coverage of 3.42% and an average depth of 1.07553706481897X.

Type: G-bacterium; Genus: *Haemophilus*, with sequence number 3,113 and relative abundance 96.32%; Species: *H. influenzae*, with sequence number 952, coverage 3.42%, and confidence level 99%, as shown in Figure 1.

#### Imaging

**Figure 2:**
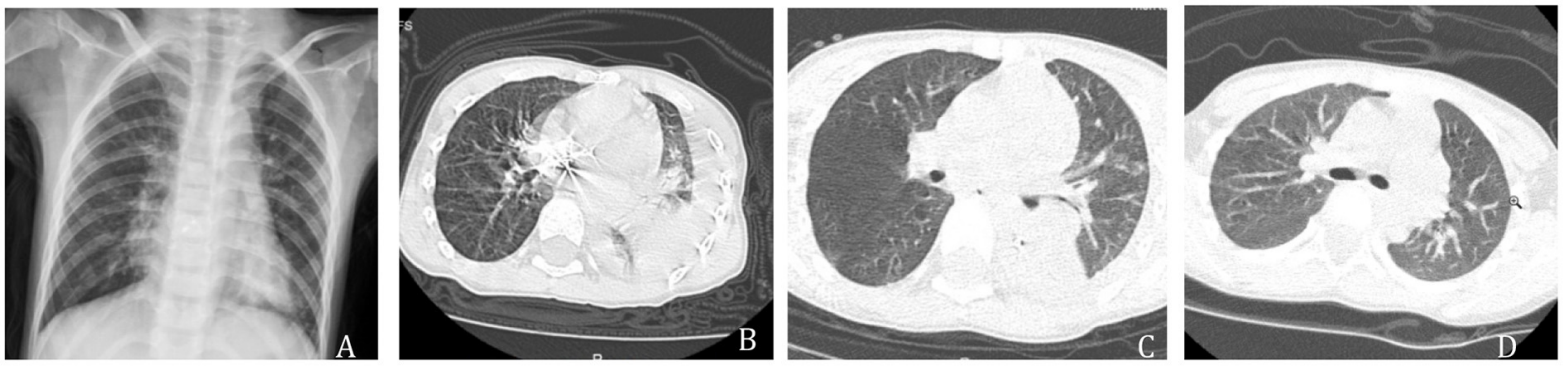
**(A)** Pre-admission outpatient chest X-ray showed inflammation in both lungs. **(B)** On admission, chest CT showed inflammation in both lungs, partial atelectasis in the left lower lobe, and left pleural effusion. **(C)** Three days after closed chest drainage, a chest CT showed localized progression of inflammation in both lungs, partial atelectasis in the left lower lobe, and resorption of left pleural effusion. **(D)** Eighteen days after antibiotic treatment, chest CT showed improvement of the inflammation in both lungs, and a small amount of pleural effusion on the left side.

The pre-admission outpatient chest X-ray revealed infiltration of both lungs. Chest and abdomen computed tomography (CT) on admission showed infiltration of both lungs, partial atelectasis of the left lower lobe, left pleural effusion, as well as more pneumatization and contents of the abdominal intestine, and a small amount of pelvic effusion. After 3 d of closed drainage of the chest, the chest CT was repeated and revealed that the inflammation in both lungs was more locally progressive than before, partial atelectasis had occurred in the lower lobe of the left lung, and left pleural effusion was less than before. Chest CT was repeated 18 d after antibiotic therapy and revealed that the inflammation in both lungs was better than before, and there was a small amount of pleural effusion on the left side, as shown in Figure 2. Ultrasound examination of pleural fluid (with the patient sitting) revealed the presence of a liquid dark area on the left side of the chest cavity, about 2.4 cm thick, with the poor internal transmission, dense dotted echogenic floating, and band separation, and located in the tube. On the right side, a dark area of fluid was visible in the chest cavity, with a thickness of about 0.5 cm and poor internal sound transmission.

An echocardiogram revealed sinus rhythm, and partial right bundle branch conduction block. A heart ultrasound revealed post-atrial septal occlusion without shunt at the atrial level, and widened coronary sinus, considering permanent left superior vena cava.

### Treatment

After admission, the patient was placed in a semi-recumbent position with nasal catheter oxygenation to improve ventilation, and blood pressure, ECG, and oxygen saturation were monitored. To control the infection, the antibiotics azithromycin and ceftriaxone were administered concurrently on the first day of admission. On the second day of admission, ultrasound-guided left pleural effusion drainage was performed, 350 ml of yellow turbid pleural fluid was drained; the body temperature gradually increased, and vancomycin combined with sulbactam sodium/cefoperazone sodium was administered to treat the infection. On the third day, the closed chest drainage was not smooth, and urokinase was administered for 3 d (dosage: 100,000 units diluted with 100 ml saline and injected through the chest drainage tube twice a day, 0.5 ml/kg each time); after each injection, the drainage tube was clamped closed and the body position was changed every 30 min, and the drainage tube was opened to drain the chest fluid after 4 h. A total of 100 ml of yellow purulent pleural fluid was drained from the closed chest cavity within 3 d, and the closed chest drainage catheter was removed after 1 w. On the 5th day of admission, through high-throughput sequencing of DNA pathogenic microorganisms in the pleural fluid, *H. influenza* was detected, thus, vancomycin was discontinued and sulbactam sodium/cefoperazone sodium was continued for 18 d. Ultrasound of the pleural fluid and chest CT were repeated, and the pleural fluid was absorbed.

### Literature search results

According to the literature search strategy used in this study, we found that the reports of pleural empyema in children caused by *H*. *influenzae* were few and old. No clinical cases of acute empyema due to *H. influenzae* have been reported in the last 5 years in China or abroad, and there was only one reported case of pleural empyema due to E. coli in a neonate. After admission, ultrasonography showed multi-interval pleural effusion, chest CT showed left pleural effusion, and multifocal scattered solid lesions. A pigtail catheter was inserted under ultrasound guidance to extract 2 ml of pleural fluid, and then a chest drain was left in place. On the first day of the placement, saline flushing failed to drain the pus. Then, an intra-thoracic injection of urokinase was used to clamp the catheter for 4 h, and purulent fluid with a little light red pleural fluid was drained. The closed-chest catheter was placed for 1 w and the chest drain was removed. The neonate was successfully treated with an intrapleural injection of urokinase with adequate antibiotic and closed chest drainage, avoiding invasive surgical interventions, and the patient was discharged after 24 d of hospitalization (5).

## Discussion

*H. influenzae*is one of the most common pathogens in pediatric community-acquired pneumonia (CAP) (6–8). According to a study by the Department of Respiratory Medicine, Shanghai Children's Medical Center, Shanghai, China, *H*. *influenzae* accounted for 14.3% of the detected pathogens in hospitalized CAP and was at the top of the list of Gram-negative bacteria (9). However, a study from the pediatric intensive care unit of the Second Hospital of Lanzhou University, Lanzhou, China, revealed that only one case of *H. influenza was* diagnosed among 49 cases of the pustular chest (10). The reason for the difference between those two studies is that most children with CAP had been given antibiotics before the development of empyema, thus, reducing the positivity rate of blood and pleural fluid cultures; as well as the previous belief that *H. influenzae* is a low pathogenic organism and severe infections are mostly seen in infants and children (11), which has led to the underestimation of the rate of severe infections in children older than 4 years.

The patient in this study was a preschool child with a fast-progressing and severe disease, and the pathogenic bacteria could not be identified in the early stage by conventional culture methods. Although conventional microbiological cultures are widely used in clinical practice, the low positivity rate and long culture time make it difficult to make a rapid and accurate diagnosis (12, 13). Bacterial culture is the gold standard for the diagnosis of *H*. *influenzae* infection, and the cultured specimens can include respiratory secretions, blood, pleural fluid, *etc*. The culture results of the patient were all negative after repeated blood and pleural fluid culture tests. With the development of precision techniques and their application in pediatric diagnosis and treatment, the application of high-throughput sequencing in the early stage of infectious diseases can undoubtedly improve the accuracy and sensitivity (14, 15). High-throughput sequencing involves taking the obtained read lengths of double-end sequencing, filtering the low-quality data, using comparison software to compare with human genomic sequences, removing the read lengths that match the human genetic sequence, comparing the remaining read lengths with the genomic databases of bacteria and viruses to obtain the distribution of bacterial and viral read lengths, and, ultimately, identifying the pathogens according to the matching ratio (16, 17). Therefore, for children with pleural effusions, an early line of simultaneous delivery of traditional pathogen culture and high-throughput sequencing of pleural fluid can improve the pathogen detection rate, and once the diagnosis of pus thorax is confirmed and pathogens are detected, sensitive antibiotics can be used for precise treatment of the disease. Once the pathogens are detected in the acute inflammatory exudate stage, sensitive antibiotics can be used to drain the pleural fluid as early as possible, which can avoid the transformation to chronic purulent chest disease and reduce the surgery rate.

The use of closed thoracic drainage in the early stages of the massive septic chest has been accepted both nationally and internationally, but in cases where pus has formed, fibrin is deposited, and septa have formed in the thoracic cavity and smooth drainage is usually not possible, which hinders treatment (18). Closed thoracic drainage combined with intrapleural injection of fibrinolytic enzymes is less invasive, has fewer side effects, shortens the hospitalization period, and reduces the chance of surgery in children with pleural empyema (19, 20). A dose-escalation trial of intrapleural infusion of urokinase in the treatment of complicated parapneumonic effusions or thorax abscess showed no intrathoracic or systemic bleeding events in subjects (21). Urokinase acts directly on the endogenous fibrinolytic system and its degradation products can be maintained for 12–24 h, which is consistent with the current clinical application approach: 100,000 units of urokinase, diluted with 100 ml of saline and injected through a chest drain twice daily at 0.5–1 ml/kg each time, with the tube clamped for 4 h after injection, followed by drainage of the effusion for 3 d (22). These studies provide more possibilities for patients with acute septic chest and can lead to a more precise and minimally invasive comprehensive treatment of septic chest in children.

In summary, *H. influenza* should not be neglected as a pus pathogen in preschool children. Early high-throughput sequencing of the DNA of pathogenic microorganisms in pleural fluid can improve the detection rate of pathogens compared with conventional bacterial culture methods. Ultrasound has a very high diagnostic value for pleural effusion and can be used as a safe and inexpensive way to follow up, as well as an important means to guide the closure of the drainage of the pleural cavity. Once a large pleural effusion occurs, the closure of the drainage of the chest cavity should be performed as soon as possible. In the case of pleural fluid ultrasound, suggesting a septum in the pleural fluid, closed chest drainage combined with intrapleural injection of urokinase is recommended. This treatment is safe and effective for septic chest, but there is little clinical experience with its application, and a large sample, multicenter prospective study is still needed to investigate optimal timing of urokinase use and the duration of intrapleural retention.

## Data Availability Statement

The original contributions presented in the study are included in the article/supplementary material, further inquiries can be directed to the corresponding author.

## Ethics Statement

The studies involving human participants were reviewed and approved by Medical Ethics Committee of The Affiliated Hospital of Hangzhou Normal University (approval number: 2021(E2)-KS-025). Written informed consent to participate in this study was provided by the participants' legal guardian/next of kin.

## Author contributions

LY: data collation, literature retrieval, and manuscript writing. GW: project management and manuscript revision. YZ: project supervisor, project management, and manuscript revision. All authors contributed to the article and approved the submitted version.

## Funding

This study was supported by the Ministry of Science and Technology of China (2021ZD0201705).

## Conflict of interest

The authors declare that the research was conducted in the absence of any commercial or financial relationships that could be construed as a potential conflict of interest.

## Publisher's note

All claims expressed in this article are solely those of the authors and do not necessarily represent those of their affiliated organizations, or those of the publisher, the editors and the reviewers. Any product that may be evaluated in this article, or claim that may be made by its manufacturer, is not guaranteed or endorsed by the publisher.
